# Multifunctional Voltage Source Inverter for Renewable Energy Integration and Power Quality Conditioning

**DOI:** 10.1155/2014/421628

**Published:** 2014-08-07

**Authors:** NingYi Dai, Chi-Seng Lam, WenChen Zhang

**Affiliations:** ^1^Department of Electrical and Computer Engineering, FST/ECE, University of Macau (UM), Avenida Padre Tomás Pereira Taipa, Macau; ^2^State Key Laboratory of Analog and Mixed-Signal VLSI, University of Macau (UM), Avenida Padre Tomás Pereira Taipa, Macau

## Abstract

In order to utilize the energy from the renewable energy sources, power conversion system is necessary, in which the voltage source inverter (VSI) is usually the last stage for injecting power to the grid. It is an economical solution to add the function of power quality conditioning to the grid-connected VSI in the low-voltage distribution system. Two multifunctional VSIs are studied in this paper, that is, inductive-coupling VSI and capacitive-coupling VSI, which are named after the fundamental frequency impedance of their coupling branch. The operation voltages of the two VSIs are compared when they are used for renewable energy integration and power quality conditioning simultaneously. The operation voltage of the capacitive-coupling VSI can be set much lower than that of the inductive-coupling VSI when reactive power is for compensating inductive loads. Since a large portion of the loads in the distribution system are inductive, the capacitive-coupling VSI is further studied. The design and control method of the multifunctional capacitive-coupling VSI are proposed in this paper. Simulation and experimental results are provided to show its validity.

## 1. Introduction

Microgrids are emerging as a consequence of rapidly growing distributed power generation systems and energy storage systems [1, 2]. Renewable energy sources (RES) have been integrated into the utility grid on a large scale to make the power generation more environmental friendly [3, 4]. In a typical residential application, the integrated renewable energy sources reduce the power demand from the grid. In order to achieve this goal, the grid-connected voltage source inverter (VSI) plays an important role as a power electronics interface to transfer power [5, 6].

Originally, the VSI for RES integration only transfers active power to the grid [7, 8]. However, most loads consume reactive power together with the active power. Considering the large number of power electronic loads such as adjustable-speed drives and diode-bridge rectifiers, harmonics also needs to be compensated in the low-voltage distribution system. If only active power consumption is reduced by the RES, the power quality in distribution system is subject to being deteriorated, especially the power factor and total harmonic distortion (THD). One solution is to install independent power quality conditioner, such as active power filter (APF). A more economic solution is to investigate the auxiliary functions of the VSI. Besides transferring the active power from the RES to the grid, the VSI is able to compensate reactive power and harmonics at the same time [9–12]. In the case like nighttime application of PV solar farms as STATCOM [10], the utilization ratio of the VSI is greatly increased.

The control and implementation of the multifunctional VSI for renewable energy integration and power quality conditioning have been discussed in previous work [11–14]. The VSI is usually coupled to the grid via an inductor or a LCL filter [9–14]. It is denoted as inductive-coupling VSI in this paper. Without a coupling transformer, the dc-link voltage of the inductive-coupling VSI is always higher than the grid voltage peak in order to transfer power and compensate harmonics. The high dc-link voltage increases the initial cost of the inverter. It also causes more switching losses and high current ripples.

Another group of grid-connected VSI, which is named capacitive-coupling VSI, has been used as power quality conditioners mainly for compensating reactive power and harmonics [15, 16]. It is coupled to the grid via a capacitor in series with an inductor and the total fundamental frequency impedance of the coupling branch is capacitive. The dc-link voltage of the capacitive-coupling VSI can be set much lower than grid voltage peak without affecting its performance in power quality conditioning [15–17]. In this paper, by adding active power transfer capability to the capacitive-coupling VSI, it is developed to a multifunctional VSI for renewable energy integration and power quality conditioning. The operation voltage of this VSI is kept low even if new functions are added, so that the energy stored in the dc bus, the system initial cost, and switching losses are greatly reduced.

The two multifunctional VSIs are first compared in Section 2. Both fundamental frequency power flow control capability and harmonics suppression capability are studied. The capacitive-coupling VSI is further studied in Section 3, in which its system design and control method are presented. Simulation verifications are provided in Section 4, including comparisons between the two multifunctional VSIs. A small capacity experimental prototype of the capacitive-coupling VSI is built and experimental verifications are given in Section 5.

## 2. Comparisons of the Multifunctional VSIs

### 2.1. Fundamental Frequency Power Flow Control Capability

**Figure 1:**
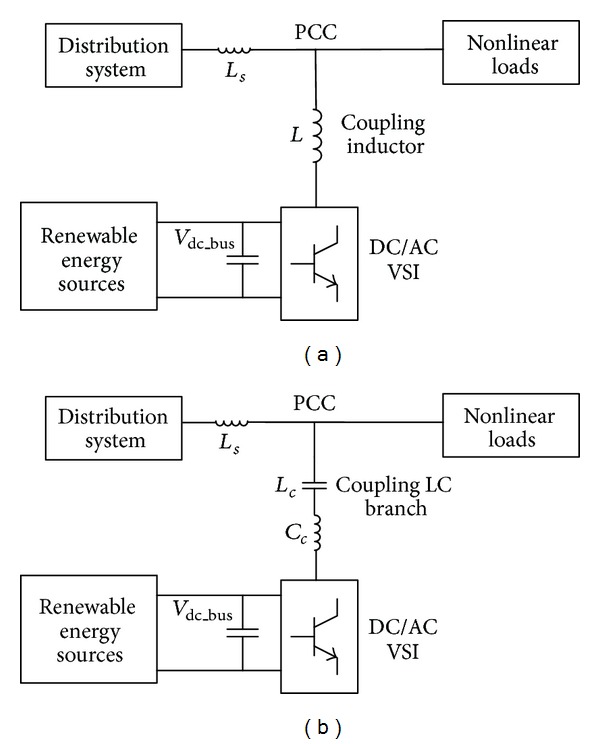
System configuration of the grid-connected VSI.

In this section, the fundamental frequency power flow control capability of the two VSIs is first analyzed. The system configurations of the two grid-connected VSIs for renewable energy integration and power quality conditioning are shown in Figure 1. The VSI is coupled to the point of common coupling (PCC) via an inductor in Figure 1(a) and it is coupled to PCC via a LC branch in Figure 1(b). The corresponding fundamental frequency equivalent circuits of these two systems are given in Table 1. In the equivalent circuit, the LC branch in the capacitive-coupling VSI is replaced by a capacitor, since the fundamental frequency equivalent impedance of this LC branch is capacitive, as given in (1). The *ω* in (1) represents the fundamental frequency in radians:

(1) $$\begin{array}{c} X_{C}=\frac{1}{\omega C}=\omega L_{C}-\frac{1}{\omega C_{C}}=X_{Lc}+X_{Cc}. \end{array}$$

If the grid-side voltage and coupling impedance are fixed, the power flow varies in terms of the operation voltage of the inverter, as expressed in [18]

(2) $$\begin{array}{c} P_{\text{inj}}=\left(\frac{V_{S}V_{\text{inv}}}{z}\cos\delta -\frac{V_{S}^{2}}{z}\right)\cos\theta +\frac{V_{S}V_{\text{inv}}}{z}\sin\delta \cdot \sin\theta \\ Q_{\text{inj}}=\left(\frac{V_{S}V_{\text{inv}}}{z}\cos\delta -\frac{V_{S}^{2}}{z}\right)\sin\theta -\frac{V_{S}V_{\text{inv}}}{z}\sin\delta \cdot \cos\theta . \end{array}$$

In (2), *V*_*s*_ is the grid voltage at the PCC; *V*_inv_ is the operation voltage of the VSI;  *δ* is the phase angle between *V*_*S*_ and *V*_inv_; *Z* and *θ* is the amplitude and angle of the coupling impedance. The power base is defined to facilitate the comparison between the two VSIs, which are expressed as follows:

(3) $$\begin{array}{c} S_{\text{base}\_L}=\frac{V_{S}^{2}}{z}=\frac{V_{S}^{2}}{\omega L}\\ S_{\text{base}\_C}=\frac{V_{S}^{2}}{z}=V_{S}^{2}\cdot \omega C. \end{array}$$

In (3), *L* indicates the coupling inductor of the inductive-coupling VSI and *C* is the equivalent impedance of the LC branch in the capacitive-coupling VSI. The same power base can be set for the two VSIs by adjusting their coupling impedance. The active and reactive power in per unit form for each VSI are listed in Table 1. The operation voltage of the VSI is calculated in terms of the active and reactive power to be transferred, and the corresponding formula is as follows:

(4) $$\begin{array}{c} \frac{V_{\text{inv}\_i}}{V_{S}}=\sqrt{{\left(\frac{P_{\text{inj}}}{S_{\text{base}\_L}}\right)}^{2}+{\left(1+\frac{Q_{\text{inj}}}{S_{\text{base}\_L}}\right)}^{2}}\\ \frac{V_{\text{inv}\_c}}{V_{S}}=\sqrt{{\left(\frac{P_{\text{inj}}}{S_{\text{base}\_C}}\right)}^{2}+{\left(\frac{Q_{\text{inj}}}{S_{\text{base}\_C}}-1\right)}^{2}}. \end{array}$$

**Figure 2:**
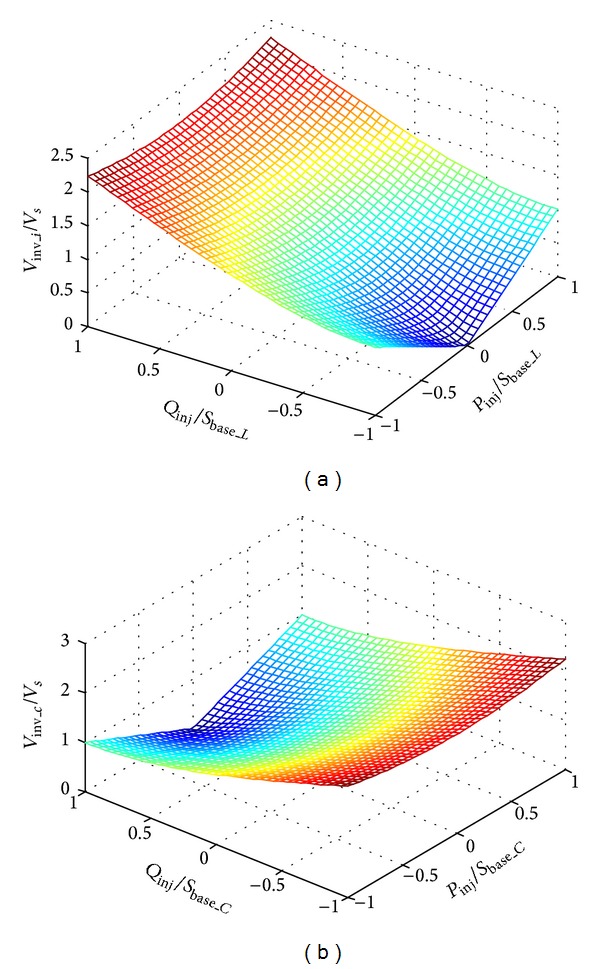
Operation voltage varies in terms of power flow.

The 3-dimensional plot of the relationship between the operation voltage and the power to be transferred is shown in Figure 2.  Figure 2(a) is for inductive-coupling VSI and Figure 2(b) is for capacitive-coupling VSI. The maximum output voltage of the inverter is limited by its dc-link voltage. The achievable power flow range increases as the operation voltage of the VSI increases. Hence the maximum power flow control capability of the VSI varies in terms of the dc-link voltage of the inverter.

**Figure 3:**
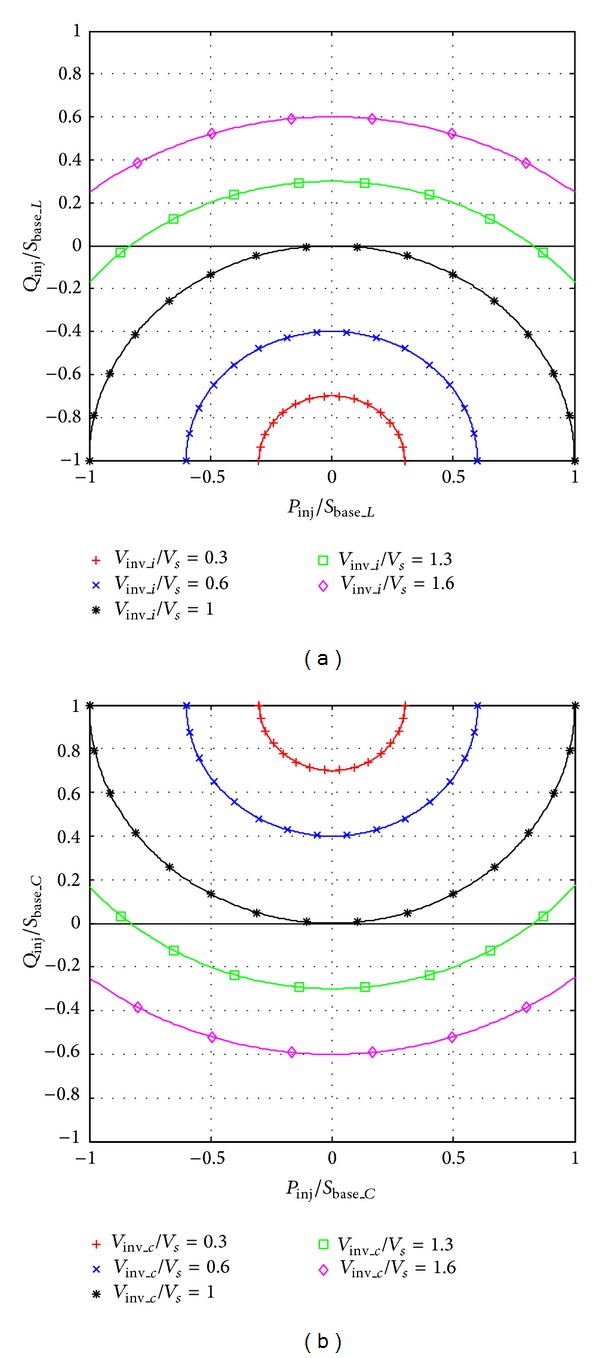
Top view of Figure 2.

The top views of the Figure 2 are given in Figure 3, which illustrates the power flow range under different operation voltage more clearly. The controllable power flow range in terms of five different operation voltages is shown in Figure 3 for both VSIs. The active power range is symmetrical about *y*-axis and increases as the voltage increases in both figures. However, the VSIs are only able to provide reactive power either positive or negative when its operation voltage in per unit is lower than one, that is, lower than the grid voltage. According to the current direction defined in the equivalent circuits, the positive reactive power corresponds to inject current lagging the grid voltage and vice versa.

In order to reduce the operation voltage, the VSI is selected in terms of the reactive power required at the PCC. For example, only positive reactive power is required for improving the power factor at the PCC, when loads are inductive. Under this circumstance, the capacitive-coupling VSI is able to transfer active power and improve the power factor simultaneously with a lower operation voltage, as illustrated in Figure 3. Hence, using capacitive-coupling VSI instead of the inductive-coupling VSI reduces the inverter voltage rating, the dc capacitor rating, and the switching losses. Although an ac capacitor is added in the coupling branch, the system total cost is lower since more can be saved by reducing inverter rating. Since a large portion of loads are inductive in the distribution system, such as motors, the multifunctional capacitive-coupling VSI is further studied in the following sections.

### 2.2. Harmonic Suppression Capability of the Two VSIs

As mentioned in previous part, more nonlinear loads are connected to the distribution system. The harmonic suppression capability is also necessary in many applications. The VSIs can reduce harmonics flowing from loads to the grid by injecting harmonic currents to the PCC. However, harmonic compensation increases the overall VSI operation voltage. When harmonic currents are injected together with the fundamental frequency current, the inverter output voltage is calculated as follows. Equation (5) is for inductive-coupling VSI and (6) is for capacitive-coupling VSI. *I*_*h*_ is the load harmonic current at *h*th harmonic. *X*_*Lh*_ and *X*_*Ch*_ are the impedance of the coupling branch at *h*th harmonics for the two VSIs, respectively:

(5) Vinv_iVS =(PinjSbase_L)2+(QinjSbase_L−1)2+Vinv_ih2 =(PinjSbase_L)2+(1+QinjSbase_L)2+∑h=2∞XLh2Ih2

(6) Vinv_cVS =(PinjSbase_C)2+(QinjSbase_C−1)2+Vinv_ch2 =(PinjSbase_C)2+(QinjSbase_C−1)2+∑h=2∞Xch2Ih2.

**Figure 4:**
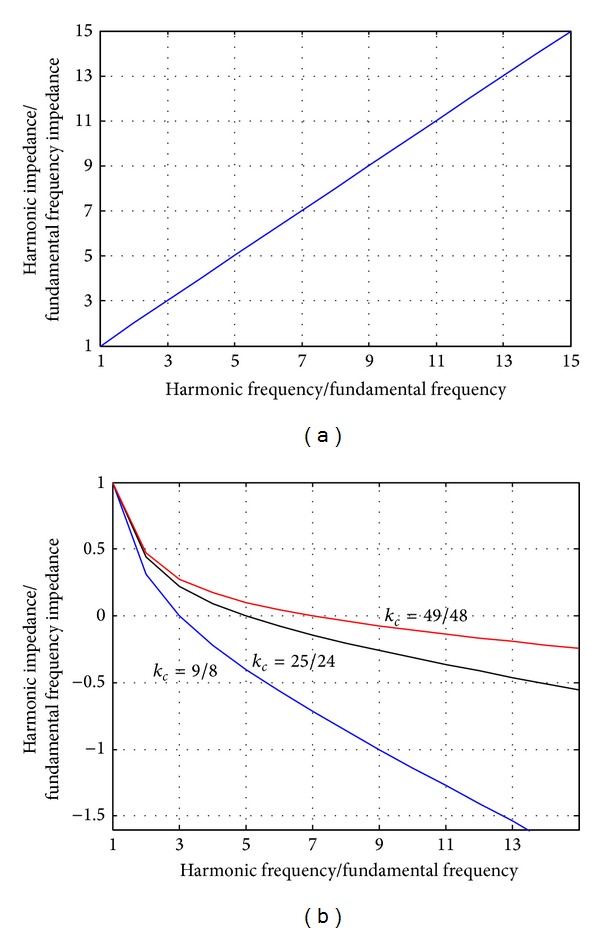
Coupling impedance varies in terms of harmonic order.

It is obvious that the impedance of the coupling branch at *h*th harmonics affects the operation voltage of the VSI. The variations of the coupling impedance in terms of frequency are given in Table 2. The impedance of the coupling inductor increases linearly proportional to the harmonic order in the inductive-coupling VSI, as shown in Figure 4(a). As a result, the operation voltage of the inductive-coupling VSI rises steeply when high order harmonics are compensated.

The coupling impedance of the capacitive-coupling VSI is the summation of the coupling inductor and capacitor. It is assumed that the impedance of capacitance *X*_*Cc*_ is as shown in (7) and inductance *X*_*Lc*_ is shown in (8):

(7) $$\begin{array}{c} X_{Cc}=k_{c}\cdot X_{C} \end{array}$$

(8) $$\begin{array}{c} X_{Lc}=\left(1-k_{c}\right)\cdot X_{C}. \end{array}$$

The impedance of the LC branch at *h*th harmonics can be expressed as (9) and illustrated in Figure 4(b):

(9) $$\begin{array}{c} X_{Ch}=X_{Lch}+X_{Cch}=\left(h+\frac{1-h^{2}}{h}\cdot k_{c}\right)X_{C}. \end{array}$$

When more than one harmonic are compensated, the value of *k*_*c*_ for minimum harmonic compensation voltage needs to be deduced. Load current harmonics are usually expressed as a percentage of fundamental current, assuming that the load harmonic current at *h*th harmonic is *r*_*h*_ times of fundamental current as expressed in

(10) $$\begin{array}{c} I_{h}=r_{h}\cdot I_{1}. \end{array}$$

The expression for determining the harmonic compensation voltage can be obtained, as shown in

(11) $$\begin{array}{c} V_{\text{inv}\_ch}^{2}=\overset{\infty }{\underset{h=2}{\sum }}{\left(h+\frac{1-h^{2}}{h}\cdot k_{c}\right)}^{2}\cdot {\left(X_{C}\right)}^{2}\cdot {\left(r_{h}\right)}^{2}I_{1}^{2}. \end{array}$$

The value of *k*_*c*_ for minimum harmonic compensation voltage can then be determined by taking the derivative of (11) with *k*_*c*_ and setting it as zero. The expression in (12) is obtained, which is used to calculate *k*_*c*_:

(12) $$\begin{array}{c} k_{c}=-\frac{\sum_{h=2}^{\infty }2\left(1-h^{2}\right)\cdot {\left(r_{h}\right)}^{2}}{\sum_{h=2}^{\infty }\left(2{\left(1-h^{2}\right)}^{2}/h^{2}\right)\cdot {\left(r_{h}\right)}^{2}}. \end{array}$$

If only harmonic at a specific frequency, that is, *n*th harmonic, is used to calculate the *k*_*c*_, (12) is simplified to

(13) $$\begin{array}{c} k_{cn}=\frac{n^{2}}{n^{2}-1}. \end{array}$$

Three cases are shown in Figure 4(b), for which the impedance of the LC branch is zero at 3rd, 5th, and 7th harmonics, respectively. There is one zero-crossing point for each curve in Figure 4(b). Correspondingly, the load harmonic at this frequency can be compensated without increasing the operation voltage of the VSI. In addition, the coupling impedance in the vicinity of the zero-crossing point is also low as shown in the Figure 4(b).

Based on above analyses, the capacitive-coupling VSI also shows advantage when harmonic suppression capability is considered. The capacitive-coupling VSI is a good alternative to serve as grid-connected VSI with active power transfer, reactive power compensation, and harmonic suppression capabilities for the low-voltage distribution system.

## 3. Design and Control of the Capacitor-Coupling VSI

### 3.1. Comprehensive Design Procedure

In this section, the comprehensive design procedure of the multifunctional capacitor-coupling VSI is proposed. The system configuration is shown in Figure 1(b).

With reference to (6), the required output voltage is minimized if reactive power to be transferred equals the power base *S*_base_*C*_. It is also validated in Figure 2(b) that the active power transfer capability of the capacitor-coupling VSI reaches peak value when its output reactive power locates in the vicinity of the *S*_base_*C*_. Hence, the capacitive-coupling VSI is better to be utilized at the PCC, whose reactive power loading varies in a small range. The average reactive power (*Q*_average_) at the PCC is used as the *S*_base_*C*_ to design the coupling impedance *X*_*C*_ at fundamental frequency.

Based on previous discussions and analysis, the detail procedure for the capacitive-coupling VSI design for minimum operation voltage under both fundamental and harmonic model is provided as follows.

(1) Select the coupling impedance according to
  (14) $$\begin{array}{c} X_{C}=\omega L_{C}-\frac{1}{\omega C_{C}}=-\frac{V_{S}^{2}}{S_{\text{base}\_C}}=-\frac{V_{S}^{2}}{Q_{\text{average}}}. \end{array}$$
(2) Calculate the coupling capacitance *Cc* according to (15), which is obtained by substituting (12) and (14) into (7):
  (15) Cc=1ω·kc·XC =1×(∑h=2∞2(1−h2)·(rh)2∑h=2∞(2(1−h2)2/h2)·(rh)2·Vs2Qaverage·ω)−1.
(3) Calculate the coupling inductance according to
  (16) Lc=(1−kc)·XCω=((1+∑h=2∞2(1−h2)·(rh)2∑h=2∞(2(1−h2)2/h2)·(rh)2)·−Vs2Qaverage) ×ω−1.
(4) Determine the dc-link operation voltage according to
  (17) Vdc=M∗2∗VS∗((PinjSbase_C)2+(QinjSbase_C−1)2  +∑h=2∞(h+1−h2h·kc)2·(Vs2Qaverage)2·(rh)2I12)1/2.

The dc-link voltage of the inverter is selected to satisfy the peak value of the inverter output voltage. The coeffcient *M* is introduced to increase the redundancy of the design. Its value usually varies between 1.1 and 1.2.

### 3.2. Control System

**Figure 5:**
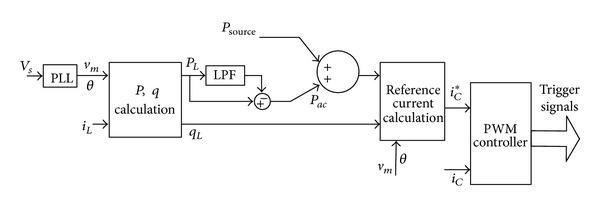
Control block diagram of the capacitive-coupling VSI.

Figure 5 shows the overall control blocks of the multifunctional capacitor-coupling VSI, in which *P*_source_ is the active power extracted from the renewable energy sources.

**Figure 6:**
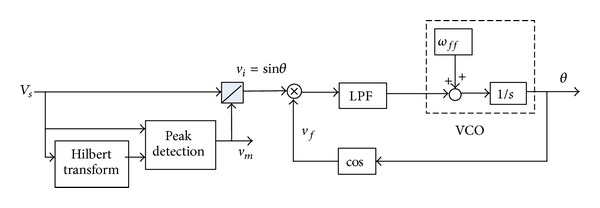
Block diagram of the software PLL.

The instantaneous reactive power theory (IRP) is used to calculate the load power [19]. To synchronize the reference current with the grid voltage, a software PLL (SPLL) is adopted in the control scheme and its block diagram is given in Figure 6 [20]. In Figure 6, the grid voltage is assumed to be *V*_*s*_ = *v*_*m*_sin*θ*. The orthogonal signal *v*_*m*_cos⁡*θ* is obtained by Hilbert transform. The peak value *v*_*m*_ of the grid voltage is calculated by

(18) $$\begin{array}{c} v_{m}=\sqrt{v_{m}^{2}{\left(\sin\theta \right)}^{2}+v_{m}^{2}{\left(\cos\theta \right)}^{2}}. \end{array}$$

The grid voltage is then divided by its peak value *v*_*m*_to get *v*_*i*_ with a unity magnitude. By multiplying *v*_*i*_ with feedback voltage *v*_*f*_, the phase error is extracted from the product by the low-pass filter. The output of the low-pass filter is sent to the voltage-controlled oscillator (VCO) to generate phase angle *θ* of the grid voltage. The feed forward signal in the VCO is *ω*_*ff*_ = 2*π*∗50 (rad/s) in a 50 Hz system.

The output of the PLL block and the peak voltage *v*_*m*_ are utilized to calculate instantaneous load power as given in

(19) $$\begin{array}{c} \left[\begin{array}{c} P_{L}\\ Q_{L} \end{array}\right]=\left[\begin{array}{c} v_{m}\sin\theta \cdot i_{L}+v_{m}\cos\theta \cdot i_{Ld}\\ v_{m}\cos\theta \cdot i_{L}-v_{m}\sin\theta \cdot i_{Ld} \end{array}\right], \end{array}$$

where *i*_*L*_ is the load current and *i*_*Ld*_ is its delay for one-fourth of a cycle. In order to inject active power from the renewable energy source, compensate reactive power, and suppress harmonics, the reference currents are calculated by

(20) ic∗=1vm2[vmsinθvmcos⁡θ][pinjqinj]=1vm2[vmsinθvmcos⁡θ][pac+psourceqL].

The reference currents are sent to the PWM unit; the output currents of the capacitive-coupling VSI are controlled to follow the reference, so that the capacitive-coupling VSI transfer active, reactive power to the grid as well as to compensate harmonics.

## 4. Simulation Results

**Figure 7:**
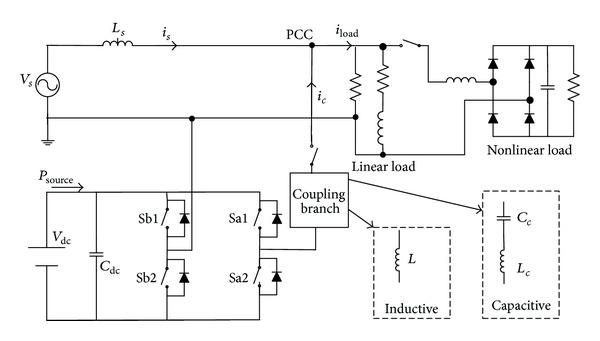
Simulation models.

Simulation models are built by using PSCAD/EMTDC. Both inductive-coupling VSI and capacitive-coupling VSI are used to achieve renewable energy integration and power quality conditioning. The system configuration is given in Figure 7, in which a single-phase VSI is used. The parameters are listed in Table 3. The renewable energy source is modeled by a dc source, which can provide active power to the dc bus of the VSI.

### 4.1. Comparison between the Two VSIs without Harmonic Compensation

**Figure 8:**
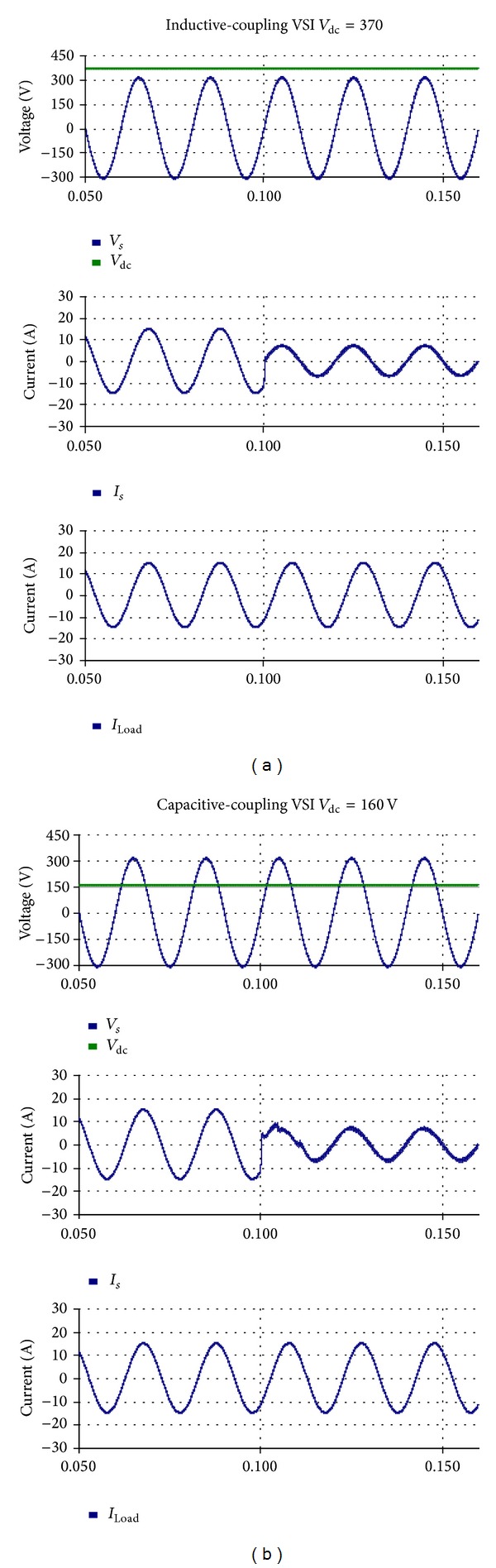
Simulation results without harmonic compensation: (a) inductive-coupling VSI and (b) capacitive-coupling VSI.

The linear loads are connected to the grid and the VSI is plugged in at 0.1 s. The two multifunctional VSIs are controlled to inject active power and reactive power to the PCC. The simulated grid voltage, dc voltage, source current, and load currents are shown in Figure 8 and the simulation results are listed in Table 4. It is obvious that the capacitive-coupling VSI achieves the fundamental frequency power transfer by using a dc-link voltage much lower than that of the inductive-coupling VSI.

### 4.2. Comparisons with Harmonic Compensation

**Figure 9:**
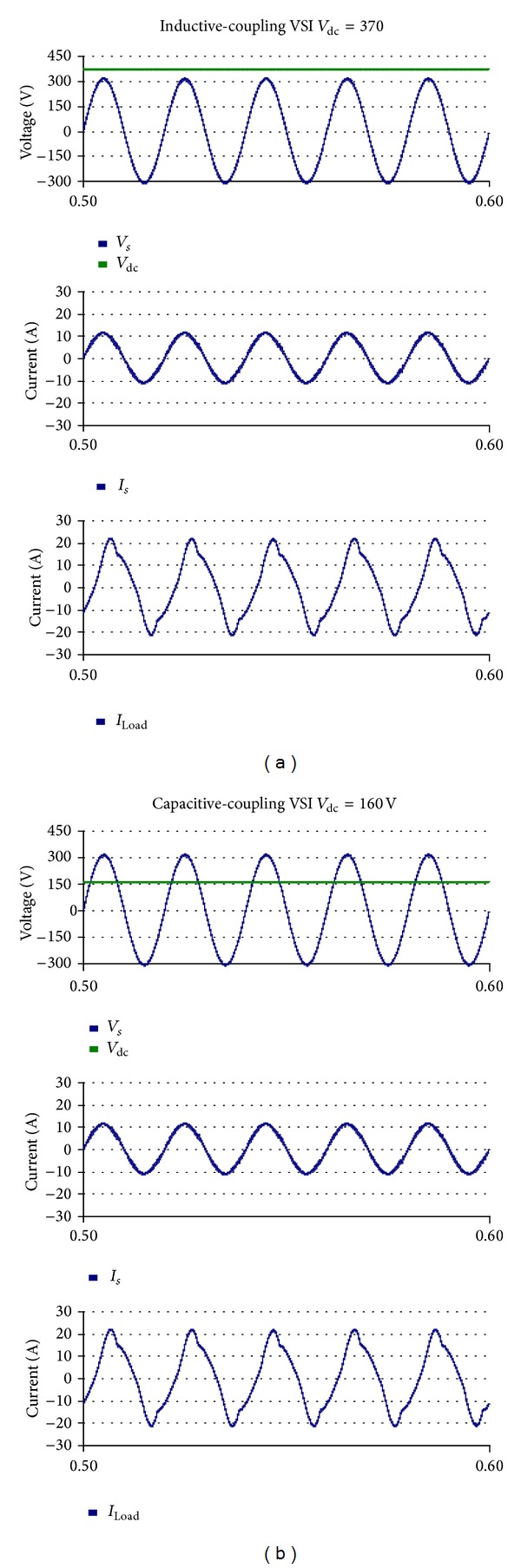
Simulation results with harmonic compensation: (a) inductive-coupling VSI and (b) capacitive-coupling VSI.

The nonlinear load is plugged in at 0.4 s. The simulation results are shown in Figure 9 and Table 5. Results indicate that the two VSIs are able to achieve renewable energy integration and power quality conditioning simultaneously. However, the operation voltage of the capacitive-coupling VSI is much lower.

## 5. Experimental Results

**Figure 10:**
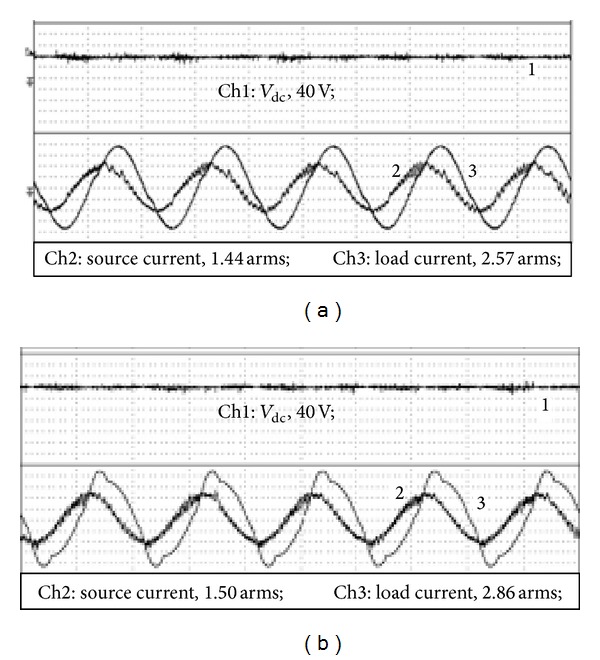
Experimental results of capacitive-coupling VSI (a) without harmonics and (b) with harmonic compensation.

A small capacity prototype of the capacitive-coupling VSI is built and the system configuration is as given in Figure 7. Due to the limitation in the laboratory, the prototype is tested by reducing the grid voltage to 55 V. Correspondingly, the dc voltage of the capacitive-coupling VSI is reduced to 40 V; the coupling impedance is the same as those in Table 3. The testing results are given in Figure 10 and Table 6. Results indicate the capacitive-coupling VSI achieves renewable energy integration and power quality conditioning with its dc-link voltage much lower than grid voltage peak. However, if an inductive-coupling VSI is used to achieve the same performance, the dc-link voltage of the VSI should be set to around 90 V.

## 6. Conclusions

In this paper, two multifunctional VSIs for renewable energy integration and power quality conditioning are studied and compared. When the capacitive-coupling VSI provides reactive power for the inductive loads, its operation voltage is much lower than that of an inductive-coupling VSI. As a result, the system initial cost and operation losses are greatly reduced. The design and control system of the capacitive-coupling VSI are presented. The simulation and experimental results are provided to show the validity of the capacitive-coupling VSI in active power transfer, reactive power compensation, and harmonics suppression.

## Figures and Tables

**Table 1:** Comparisons for fundamental frequency power flow control.

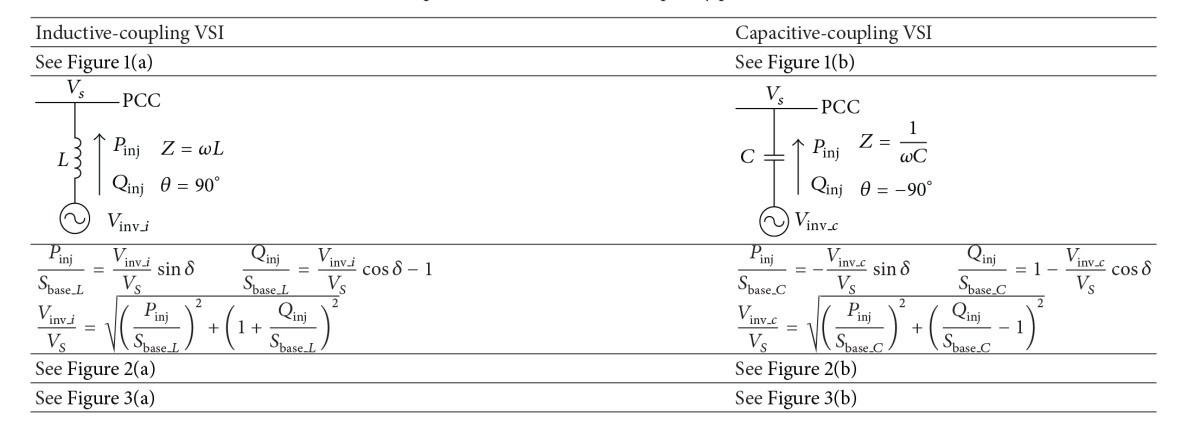

**Table 2:** Comparisons with harmonic suppression capability.

Inductive-coupling VSI	Capacitive-coupling VSI
*X*_*Lh*_ = *hωL* = *hX*_*L*_ *h*-harmonic order	$X_{Ch}=X_{Lch}+X_{Cch}=(h+\frac{1-h^{2}}{h}\cdot k_{c})X_{LC}$ *h*-harmonic order

See Figure 4(a)	See Figure 4(b)

**Table 3:** System parameters in the simulation.

System setting	
Grid voltage *V*_*s*_	220 V
Source inductor *L*_*s*_	0.1 mH

Inductive-coupling VSI	
Coupling Inductor (*L*)	8 mH
dc-link voltage (*V*_dc_)	370 V

Capacitive-coupling VSI	
Coupling capacitor (Cc)	130 uF
Coupling inductor (*L*_*c*_)	3.5 mH
dc-link voltage (*V*_dc_)	160 V

**Table 4:** Simulation results without harmonics compensation.

	RMS	Active power	Power factor	Current THD
Linear load current	10.32 A	1525 W	0.66	0.1%
Grid side (inductive-coupling VSI)	5.51 A	1062 W	1.00	2.81%
Grid side (capacitive-coupling VSI)	5.59 A	1058 W	1.00	2.74%

**Table 5:** Simulation results with harmonics compensation.

	RMS	Active power	Power factor	Current THD
Nonlinear load current	13.5 A	2162 W	0.74	15.3%
Grid side (inductive-coupling VSI)	7.76 A	1707 W	1.00	2.75%
Grid side (capacitive-coupling VSI)	7.73 A	1695 W	1.00	2.00%

**Table 6:** Experimental results of capacitive-coupling VSI.

	Current RMS	Active power	Power factor	Current THD
Linear load current	2.57 A	90 W	0.64	—
Source current	1.44 A	72 W	0.98	5.91%
Nonlinear load current	2.86 A	99 W	0.63	14.2%
Source current	1.50 A	79 W	0.98	6.61%
